# 2365: PTSD: Understanding differences in trauma cognitions, memory, and emotional regulation

**DOI:** 10.1017/cts.2017.265

**Published:** 2018-05-10

**Authors:** Ellen Volpe, Tiffany Jenzer, Lauren Rodriguez, Jennifer P. Read

## Abstract

OBJECTIVES/SPECIFIC AIMS: Low-income, urban adolescents experience high rates of interpersonal and community violence and consequently post-traumatic stress disorder (PTSD). Memory theory purports that the development of PTSD can be explained by dysfunctional trauma cognitions, high sensory and poor articulation of trauma memories, and poor emotional regulation. The purpose of this paper are as follows: (1) to describe trauma experiences and PTSD symptoms of a high-risk sample of low-income urban youth and (2) to explore if post-traumatic cognitions, trauma memory quality, or emotional regulation means differ in participants screening positive for PTSD as compared with those that did not screen positive. METHODS/STUDY POPULATION: A preliminary sample of low-income, urban adolescents (ages 16–21) at risk for homelessness took a web-based, self-report survey responding to questions related to their experiences of trauma and mental health (n=48). PTSD was measured with the PTSD Checklist for DSM-5 criteria (PCL_5). A cut-off of 33 was used as a positive screen for PTSD. Post-traumatic cognitions was measured with the post-traumatic cognition inventory (pcti) with higher scores representing greater dysfunctions and negative cognitions. Trauma memory was measured with the Trauma Memory Quality Questionnaire (TMQQ) with higher scores representing more sensory-based and poorly verbalized memories. Emotional regulation was measured using the Difficulties in Emotional Regulation Scale (DERS) with higher scores representing greater difficulties with emotional regulation. All 3 variables conceptually represented theoretical constructs of the development of PTSD. Initial data from the baseline survey was used conducted a 1-way ANOVA to compare the difference in post-traumatic cognition, quality of trauma memory, and emotional regulation in those that screened positive for PTSD as compared with their peers. RESULTS/ANTICIPATED RESULTS: The majority of this population (80%) experienced at least 1 traumatic life event. This sample experienced an average of 10.5 lifetime traumas (SD=10.6). Of those experiencing trauma about 20% (n=8) reported a positive PTSD screen (PCL-5). There were significant group differences among those screening positive for PTSD and their peers in the following variables: (1) pcti (*F*_1,24_=10.43, *p*<0.004), (2) TMQQ (*F*_1,29_=11.02, *p*<0.002), and (3) DERS (*F*_1,36_=19.68, *p*=0.000). The majority of this population (80%) experienced at least one traumatic life event. This sample experienced an average of 10.5 lifetime traumas (SD=10.6). Of those experiencing trauma about 20% (N=8) reported a positive PTSD screen (PCL-5). There were significant group differences among those screening positive for PTSD and their peers in the following variables: 1) pcti [F(1,24) = 10.43, p<.004], 2) TMQQ [F(1,29) =11.02, p<.002], [F(1,36) =19.68, p=.000]. DISCUSSION/SIGNIFICANCE OF IMPACT: This sample reported high rates of trauma and PTSD. Constructs representing memory theory (cognition dysfunction, quality of memory, and emotional regulation) all significantly differed among participants with PTSD compared with their peers. Consequently, it may be useful for trauma interventions to target the maladaptive post-traumatic cognitions, quality of traumatic memories, and emotional regulation in this population. These results will inform work that aims to explore if a trauma intervention, based on the memory theory can improve PTSD symptoms. Anticipated data collection completion in March 2017 (n=120).

